# A survey on the institutional practices of the use of isavuconazole in onco-hematological children, on behalf of the Infectious Working Group of AIEOP

**DOI:** 10.1007/s00431-026-07432-1

**Published:** 2026-09-23

**Authors:** Lorenzo Chiusaroli, Manuela Spadea, Milena La Spina, Letizia Pomponia Brescia, Maria Rosaria D’Amico, Erica Ricci, Maria Grazia Petris, Marica De Pieri, Katia Perruccio, Alessia Pancaldi, Paola Muggeo, Antonella Colombini, Federica Galaverna, Pietro Gasperini, Valeria Petroni, Elena Soncini, Simona Rinieri, Francesca Compagno, Rossella Mura, Francesco Baccelli, Angelica Barone, Maria Vittoria Micheletti, Alessandra Biffi, Daniele Donà, Simone Cesaro

**Affiliations:** 1 Division of Pediatric Infectious Diseases, Department for Women’s and Children’s Health, University of Padua, Padua, Italy; 2 Stem Cell Transplantation and Cellular Therapy Division, Pediatric Onco-Hematology, Regina Margherita Children’s Hospital, Turin, Italy; 3 University of Turin, Turin, Italy; 4 Pediatric Hematology and Oncology Unit, Azienda Ospedaliero Universitaria Policlinico “G. Rodolico - San Marco”, Catania, Italy; 5 Hemato-Oncology Unit, SS Annunziata Hospital, Taranto, Italy; 6 UOC TCE and Cellular Therapies, Department of Pediatric Hematology-Oncology and Cellular Therapies, Santobono-Pausilipon, Naples, Italy; 7 Division of Infectious Diseases, IRCCS Istituto Giannina Gaslini, Via Gerolamo Gaslini 5, Genoa, Italy; 8 Hematology and Oncology Unit, Department for Women’s and Children’s Health, University Hospital of Padua, Padua, Italy; 9 Pediatric Oncology Hematology, Mother and Child Health Department, Santa Maria Della Misericordia Hospital, Perugia, Italy; 10 Pediatric Unit, Azienda Ospedaliero-Universitaria Policlinico, Modena, Italy; 11 U.O.C. Pediatria Ad Indirizzo Oncoematologico Azienda Ospedaliero Universitaria Consorziale Policlinico, Bari, Italy; 12 Department of Pediatrics, Fondazione MBBM - Ospedale San Gerardo, Monza, Italy; 13 Department of Pediatric Oncology, Cell and Gene Therapy, IRCCS Ospedale Pediatrico Bambino Gesù, HematologyRome, Italy; 14 Department of Pediatrics, Azienda Unità Sanitaria Locale Di Rimini, Rimini, Italy; 15 Division of Pediatric Hematology and Oncology, Ospedale G. Salesi, Ancona, Italy; 16 Pediatric Oncohematology and Bone Marrow Transplant Unit, Children’s Hospital, Spedali Civili, Brescia, Italy; 17 DH Pediatric Oncology Hematology, University Hospital S. Anna, Ferrara, Italy; 18 Pediatric Hematology/Oncology Unit, Fondazione IRCCS Policlinico San Matteo, Pavia, Italy; 19 Pediatric Hematology Oncology, Azienda Ospedaliera Brotzu, Cagliari, Italy; 20 Pediatric Oncology and Hematology Unit “Lalla Seràgnoli”, Pediatric Unit, Istituto Di Ricovero E Cura a Carattere Scientifico (IRCCS), Azienda Ospedaliero-Universitaria Di Bologna, Bologna, Italy; 21 Pediatric Onco-Haematology, Azienda Ospedaliero-Universitaria Di Parma, Parma, Italy; 22 Pediatric Hematology Oncology, Bone Marrow Transplant, Azienda Ospedaliera Universitaria Pisana, Pisa, Italy; 23 Pediatric Hematology Oncology, Department of Mother and Child, Azienda Ospedaliera Universitaria Integrata, Verona, Italy

**Keywords:** Isavuconazole, Fungal infections, Pediatric, Children, Oncology, Hematology, Antifungal

## Abstract

Isavuconazole is a broad-spectrum antifungal agent approved for the treatment of invasive fungal infections (IFIs) in adults and, recently, in pediatric patients aged 1 year and older. Use in the pediatric setting remains limited, but available data indicate generally favorable safety and efficacy. This survey describes the institutional practices of use of isavuconazole across centers belonging to the Italian Association of Pediatric Hematology and Oncology (AIEOP). A 25-item web-based questionnaire, approved by the scientific board of the Infectious Working Group, was distributed to AIEOP centers between December 2024 and January 2025. The survey covered indications (prophylaxis and treatment), formulations and dosing, therapeutic drug monitoring (TDM), drug–drug interactions, and management of adverse events. In 22/49 (45%) responding centers, isavuconazole was available as capsules in 20/22 (91%) and as an intravenous formulation in 17/22 (77%). Primary prophylaxis was reported in 2/22 (9%), while secondary prophylaxis was reported in 12/22 (55%) centers, in patients diagnosed with possible (10/22, 45%) or probable (12/22, 55%) IFIs. In cases of *Aspergillus* spp. infection, 4/22 (18%) centers considered isavuconazole for first-line treatment, 14/22 (64%) for salvage treatment, and 5/22 (23%) for combination treatment. In cases of mucormycosis, 4/22 (18%) considered isavuconazole for first-line treatment, 9/22 (41%) for salvage treatment, and 5/22 (23%) for combination treatment. TDM was commonly adopted but varied across centers: 7/22 (32%) performed TDM between days 5 and 7, 4/22 (18%) only after the loading dose, and 5/22 (23%) did not perform TDM.

*Conclusion*: The results of this study show a widespread, off-label, heterogeneous use of isavuconazole in AIEOP centers. Such variability indicates the need for more standardized indications, dosages, and monitoring strategies.

**Table:** 

What is known? • *Isavuconazole is approved for invasive fungal infections in adults and, recently, in children aged =1 year, with favorable safety and efficacy, although pediatric experience remains limited.* • *Real-world use of isavuconazole in pediatric hematology-oncology has not been systematically described.*
What is new? • *This national AIEOP survey shows widespread but heterogeneous use of isavuconazole, mainly as secondary prophylaxis and salvage therapy, with inconsistent therapeutic drug monitoring.* • *Standardized indications, dosing, and monitoring strategies are needed in pediatric onco-hematology.*

**Supplementary Information:**

The online version contains supplementary material available at https://doi.org/10.1007/s00431-026-07432-1.

## Background

Invasive fungal infections (IFIs) represent a significant cause of morbidity and mortality in immunocompromised pediatric patients, particularly those with hematologic malignancies, undergoing hematopoietic cell transplantation (HCT), or receiving intensive immunosuppressive therapy. The management of IFIs in children poses unique challenges due to developmental pharmacokinetics, limited therapeutic options, and a lack of robust pediatric-specific data [1]. Triazoles have become a cornerstone of antifungal therapy. Although they carry a risk of drug–drug interactions, this class is generally well tolerated. Most agents offer broad antifungal coverage and demonstrate clinical effectiveness across a wide range of indications, depending on the specific fungal pathogen, the clinical context, and the approved uses of each individual drug [2].

Among these, isavuconazole, a broad-spectrum triazole, has demonstrated efficacy and a favorable safety profile in the treatment of IFIs in adult populations. Its once-daily dosing, availability in both intravenous and oral forms via the water-soluble prodrug isavuconazonium sulfate, and predictable pharmacokinetics make it a promising option for pediatric patients [3].

Compared with other anti-mold azoles, isavuconazole demonstrates a more predictable TDM profile, fewer drug–drug interactions than posaconazole, and fewer adverse events than voriconazole in the pediatric population [4, 5]. However, experience with isavuconazole in the pediatric setting remains limited, with most current knowledge derived from adult studies and small pediatric observational studies [6–8]. Isavuconazole received regulatory approval in Europe for patients aged over 1 year for the treatment of *Mucorales* and *Aspergillus* infections [9]. Nevertheless, there are several issues regarding its use including dosing, therapeutic drug monitoring (TDM), and safety that would deserve practice harmonization and further investigation. This survey aims to define the scenario of the use of isavuconazole in the centers belonging to the Italian Association of Pediatric Hematology and Oncology (AIEOP). We report the results of a national pediatric survey.

## Methods

The survey was conducted among the 49 centers of the Infectious Diseases Working Group (IDWG) of AIEOP with the aim of assessing the use of isavuconazole.

A 25-item web-based questionnaire was developed by the scientific committee of IDWG-AIEOP comprising pediatric hematologist-oncologists, infectious diseases specialists, and pediatric transplanters. The questions were developed with reference to the European Conference on Infections in Leukemia (ECIL) 8 recommendations and in conjunction with discussion among the investigators, who have extensive experience in antifungal therapy and fungal disease in immunocompromised children. Usability testing for the survey was conducted at two pilot hospitals, selected to ensure generalizability. Positive feedback was received regarding the ease of use and time taken to complete the survey; therefore, no changes were made.

The questionnaire was sent to all AIEOP centers between December 16, 2024, and January 30, 2025. A link to the secure online survey (REDcap®), was sent via email to all nominees on 15 December 2024, and data submission closed on 31 January 2025. A weekly recap email was sent until the submission closed. In each center, the questionnaire was completed by the local IDWG-AIEOP nominee, generally the physician responsible for infectious disease management within the pediatric hematology-oncology or transplant unit and involved in defining internal antimicrobial protocols. All questions were mandatory and completed by every respondent. Since it was a self-reported survey, answers were checked for inconsistencies to enhance data accuracy; if any discrepancies were noticed, a researcher would contact the respondent for clarification. Hospital data were anonymized before reporting, and participation in the survey was voluntary. Data were analyzed using descriptive statistics. The results aimed to characterize availability and formulations, indications for prophylaxis (primary/secondary) and treatment (empirical, possible, probable/proven IFI), dosing regimens, TDM timing and targets, and adverse event management.

## Results

The center’s response rate was 22/49 (45%), including 12 II level hospital and 10 III level pediatric hospital, and 41% (9/22) of them were accredited for the pediatric transplant activity. The centers were in 13 of 20 administrative regions of Italy, enabling an almost nationwide assessment. The complete questionnaire and collected responses are presented in Table 1. The results of this survey are compared with the pediatric indications of ECIL recommendations in Supplementary material Table S1.

**Table 1:** Complete survey questionnaire and responses

Questions	Responses
Which formulations of isavuconazole are available in your institution?	
Infusion solution	17/22
Hard capsules	20/22
Which posology do you use?	
5.4 mg/kg three times daily for the first 2 days and following once daily	14/22
10 mg/kg three times daily TDM driven	2/22
Individualized posology	6/22
Do you use the isavuconazole for the antifungal prophylaxis?	
Yes	2/22
No	0/22
For which disease do you use?	
ALL high-risk	0/22
Relapse ALL	0/22
AML	1/22
Allogenic HCT	2/22
Autologous HCT	0/22
Aplastic anemia	0/22
Do you use, or have you used isavuconazole for secondary prophylaxis after a possible or probable invasive fungal infection?	
Possible	10/22
Probable	14/22
Do you use, or have you used isavuconazole as empirical antifungal treatment in patients with fever, neutropenia and with a history of fungal infection?	
Yes	5/22
No	17/22
Do you use, or have you used isavuconazole as an antifungal treatment in patients with invasive fungal infection possible and probable with or without previous fungal infection?	
Possible without previous infection: yes, not as first-line therapy	9/22
Probable without previous infection: yes, not as first-line therapy	9/22
Possible with previous infection	
Yes, as first-line therapy	12/22
Yes, not as first-line therapy	12/22
Probable without previous infection	
Yes, as first-line therapy	12/22
Yes, not as first-line therapy	12/22
In the patient with persistent fever and neutropenia, in prophylaxis with isavuconazole, how do you behave if the patient is febrile after 72–96 h of broad-spectrum antibiotic?	
Discontinue the prophylaxis and start empirical antifungal therapy	2/22
Repeat the diagnostic check-up and decide based on this	2/22
How do you consider the use of isavuconazole in *Aspergillus* spp. infections?	
First-line therapy	4/22
Salvage therapy or second line	14/22
Combined therapy	5/22
How do you consider the use of isavuconazole in *Mucor* spp. infections?	
First-line therapy	4/22
Salvage therapy or second line	9/22
Combined therapy	5/22
When do you check the first serum level after starting isavuconazole?	
Between 5 and 7 days	7/22
After 7 days	2/22
Do not check	5/22
Check after 48 h (loading dose)	4/22
How often do you repeat the serum level (TDM) of isavuconazole after reaching the optimal level?	
Once every 2 weeks	4/22
Once weekly	2/22
Once monthly	1/22
How do you manage the isavuconazole adverse events?	
Discontinue isavuconazole	9/22
Continue using isavuconazole while monitoring its threshold level	7/22

*TDM*, therapeutic drug monitoring; *ALL*, acute lymphoblastic leukemia; *AML*, acute myeloid leukemia; *HCT*, hematopoietic stem cell transplantation

### Formulations and dosage

All 22 centers reported having access to isavuconazole. Capsules were available in 20/22 (91%), and the intravenous (IV) formulation in 17/22 (77%). Regarding the posology, different administration schedules had been adopted by centers, as follows: 5.4 mg/kg three times daily for the first 2 days and following once daily in 14/22 (64%), 10 mg/kg three times daily TDM driven in 2/22 (9%), and 6/22 (27%) centers declared generally to adopt a patient personalized posology.

### Treatment

Five out of 22 (23%) centers reported using isavuconazole, particularly in cases of prior IFI. Instead, clinicians tended to prescribe isavuconazole in cases of possible or probable IFI, both with (12/22, 54%) and without (9/22, 41%) a history of IFI (Supplementary material, Fig. S1).

Regarding management of a suspected breakthrough IFI during isavuconazole prophylaxis, 9% of centers had maintained prophylaxis, but performed diagnostic testing, with subsequent decisions based on the results of diagnostic testing. Instead, 9% continued the prophylaxis with isavuconazole, monitoring the patient’s clinical condition. In the treatment of invasive aspergillosis, 4/22 (18%) centers reported using isavuconazole for first-line therapy, 14/22 (64%) for salvage therapy, and 5/22 (23%) in combination therapy. In the treatment of mucormycosis infections, isavuconazole was used as first-line therapy in 4/22 (18%) centers, as salvage therapy in 9/22 (41%), and as combination therapy in 5/22 (23%).

### Prophylaxis

Primary prophylaxis with isavuconazole was administered only in 2/22 (9%) centers and, of them, only in 1/22 (5%) centers in AML patients. Among the 9 centers performing allogeneic HCT, only 2/9 used isavuconazole as primary prophylaxis.

None of the centers declared to use isavuconazole for ALL, aplastic anemia, or other hematological disease. Most centers used isavuconazole as secondary prophylaxis (12/22, 55%) in patients with previous possible and probable IFI in 10/22 (45%) and 12/22 (55%) of cases, respectively (Supplementary materials, Fig. S2).

### TDM and interactions

The methods for conducting the initial TDM assessment varied across centers: 7/22 (32%) performed it between 5 and 7 days; 2/22 (9%) did it after 7 days; and 5/22 (23%) did not perform TDM at all. In 4/22 (18%) centers, TDM was done after the loading dose, which was 48 h. Follow-up TDM measurements occurred every 2 weeks in 4/22 (18%) centers, weekly in 2/22 (9%) centers, and monthly in 1/22 (5%) centers. One center only checked TDM once, following the loading dose. Management of adverse events differed among centers: 9/22 (41%) centers withdrew isavuconazole and switched to an alternative antifungal, while 7/22 (32%) centers reduced the dose and continued treatment after obtaining TDM results.

## Discussion

The results of this survey show that isavuconazole is widely available but used heterogeneously. The primary use is as salvage therapy for invasive aspergillosis (14/22, 64%), whereas use as first-line treatment for IFI is limited, reflecting the relatively recent pediatric approval. On the other hand, isavuconazole is more commonly used in mucormycosis, including first-line, salvage, and combination therapy, consistent with adult practice [10].

The evidence of therapeutic use in children remains limited to small cohorts. A multicenter series, comprising 24 patients reported an overall response rate of ~ 70%, including combination regimens (administered in 7 patients) [11].

Other studies reported smaller cohorts but showed comparable response rates [12, 13].

Isavuconazole is also infrequently administered in the setting of primary prophylaxis, particularly considering the poor compliance with oral prophylaxis in younger children and in those with post-chemotherapy mucositis or enteritis. Indeed, only 2/22 (9%) centers in our survey reported employing it for this indication. By contrast, the use of isavuconazole for secondary prophylaxis was more common. The literature data on isavuconazole prophylaxis in children remain limited but encouraging in the largest pediatric series reported to date; 3 cases of breakthrough invasive fungal infections were observed among 20 patients receiving primary prophylaxis [14]. Recently, Mendoza-Palomar et al. described the largest pediatric population to receive isavuconazole for both treatment and prophylaxis: among patients treated for proven/probable IFI, an overall clinical response rate of 60% was observed, while the rate of adverse events was 25%. They also reported that 12 patients received isavuconazole as primary prophylaxis, and no IFI occurred [8].

The role of isavuconazole in the primary prophylaxis setting is still under investigation, including in adult patients.

In ECIL guidelines, isavuconazole receives a weak recommendation (C-III) in adult patients with acute lymphoblastic leukemia (ALL) and a moderate strength of evidence (B-II) in those with acute myeloid leukemia (AML) [15].

The need for TDM in the pediatric setting remains undefined. Adult trials report serum concentrations typically within 1–7 mg/L, and the value of TDM remains debated [16].

Our findings mirror this uncertainty: most centers perform TDM but with diverse timing and frequency, and nearly a quarter do not monitor levels, while 7/22 (32%) centers in our survey reported adopting TDM strategies based on liver enzyme elevation.

The considerable heterogeneity observed in dosing strategies and in the positioning of isavuconazole relative to other antifungal agents likely reflects the absence of pediatric-specific dosing guidelines and the recency of regulatory approval in this population, which leaves individual centers to extrapolate adult protocols or rely on institutional experience. The choice between isavuconazole, voriconazole, and posaconazole may be further influenced by drug availability and formulary restrictions, local costs and reimbursement policies, familiarity with each agent’s toxicity and interaction profile, and the need to avoid azole cross-resistance in patients with prior antifungal exposure. Isavuconazole’s more favorable drug–drug interaction profile and simpler oral-to-intravenous transition make it particularly advantageous for patients undergoing intensive immunosuppressive or chemotherapy regimens. Conversely, extensive clinical experience in pediatric populations may lead other centers to prefer posaconazole or voriconazole as first-line options [17, 18].

Center-level characteristics may also contribute to this variability. Larger centers and those with higher transplant volumes are more likely to have in-house or rapidly accessible TDM services, dedicated infectious disease expertise, and standardized institutional protocols, all of which could support more consistent, protocol-driven use of isavuconazole. Conversely, centers with lower case volumes or without ready TDM access may adopt more individualized, empirical approaches, consistent with the high proportion of centers reporting personalized posology in our survey. Although our survey did not collect data on individual center characteristics such as size, transplant activity, or TDM availability, exploring these associations in future multicenter studies could help clarify whether the observed heterogeneity reflects rational adaptation to local resources or an unmet need for shared, pediatric-specific practice guidelines [19].

Overall, the favorable tolerability and drug–drug interaction profile of the compound, together with dual oral/intravenous availability, make it a pragmatic option across pediatric scenarios.

However, due to the nature of the survey, our study has several limitations. We did not collect patient-level data, but rather focused on the application of clinical practices in routine settings. As a result, certain aspects could not be fully explored, such as the specific contexts guiding the choice between capsule and intravenous formulations, detailed information on TDM sampling, and comprehensive data on underlying conditions. In addition, the response rate was 22/49 (45%) across all AIEOP affiliates, representing the main active centers but not all facilities nationwide. In conclusion, our survey highlights several challenges associated with the use of isavuconazole, including uncertainties regarding its optimal clinical positioning, limited pediatric indications, and the potential need for TDM. Particular attention should be directed to these issues, and additional information is expected from the ongoing trial [20]. Prospective pediatric studies and consensus guidelines are warranted to refine indications, define exposure targets, and harmonize monitoring approaches.

## Supplementary Information

Below is the link to the electronic supplementary material.

Supplementary file1 (DOCX 227 kb)

## Data Availability

The original contributions presented in this study are included in the article. Further inquiries can be directed to the corresponding author.
