# Supplementary material for: A survey on the institutional practices of the use of isavuconazole in onco-hematological children, on behalf of the Infectious Working Group of AIEOP
Source: Eur J Pediatr. 2026 Sep 23;185(10):772. doi: 10.1007/s00431-026-07432-1 (PMC13597545; doi:10.1007/s00431-026-07432-1)
Supplement: Supplementary file 1 [file 431_2026_7432_MOESM1_ESM.docx]

**Supplementary Materials**

**Table S1. Isavuconazole Survey findings compared with the European Conference on Infections in Leukemia (ECIL) recommendations.**

| **Domain** | **Recommendation** | **Survey findings** | **Appraisal** | **Comments** | **Implication for practice** |
| --- | --- | --- | --- | --- | --- |
| **Primary prophylaxis – AML** | Moderately supported in adults, but no approval for prophylaxis of IFD. Not supporting data for children | Used in 1/22 (5%) centers | Aligned, but evidence base limited | Reflect clinicians’ preferences other routinely used, easily available and manageable drugs | Understanding the indication in national pediatric specific guidelines |
| **Primary prophylaxis – ALL (including relapse)** | Low evidence in adults, not supporting data for children. | 0/22 | Aligned | Reflect clinicians’ not clearly application | Understanding the indication in national pediatric specific guidelines |
| **Primary prophylaxis – allogeneic HCT** | Supported in adults, pre-engraftment and post engraftment GVHD. Not supporting data for children | Used in 2/9 (22%) HCT centers | Partially aligned | Reflect clinicians’ preferences other routinely used, easily available and manageable drugs | Understanding the indication in national pediatric specific guidelines |
| **Secondary prophylaxis** | Case-by-case in pediatrics | Used in 10/22 (45%, possible); 12/22 (55%, probable) | Generally consistent, evidence base limited | Reflect clinicians’ preferences and experience | Understanding the indication in national pediatric specific guidelines |
| **Formulations** | IV for predictable exposure; Hard capsules difficult administration <5 years | Capsules available in 20/22 (91%); IV formulation available in 17/22 (77%). | IV preferable for loading dose or in younger children; hard capsules may be difficult to administer in this group. | Limited application for younger outpatients | Work with pharmacy/health authorities for possible more applicable formulations. |
| **Dosing – Hard capsules/ Intravenous** | Weight-banded per label/guidelines | 14/22 (64%) standard loading followed by maintenance dose; 2/22 (9%) TDM-driven dosing; 6/22 (27%) personalized dose | Heterogeneous; many unspecified | Reflects heterogeneity of dosing strategies. | Standardize dosing in national pediatric specific guidelines |
| **PK -TDM first level** | Adult data suggested stable PK, not clear indication with check | Four different schedules reported: 7/22 (32%) between days 5–7; 2/22 (9%) after day 7; 4/22 (18%) after the loading dose; 5/22 (23%) did not perform TDM at all. | Not clear indication in children | Not clear indication in children and reflect limited resources (TDM not available in the same hospital) | Standardize indication and ensure funding and lab access for timely assays |
| **PK- TDM maintenance** | Adult data suggested stable PK, not clear indication with check | 4/22 (18%) q2w, 2/22 (9%) weekly, 1/22 (5%) monthly | Not clear indication in children | Not clear indication in children and reflect limited resources (TDM not available in the same hospital) | Standardize indication and ensure funding and lab access for timely assays |
| **DDI and adverse events** | No particularly concerns for DDI | 9/22 (41%) withdrew isavuconazole and switched to an alternative antifungal; 7/22 (32%) reduced the dose | Not clear data in children population. Safety data promising | Reflect center-specific policy | Build a prospective registry on adverse events management |

The table summarizes the European Conference on Infections in Leukemia (ECIL) 8 and 10 recommendations concerning the use of isavuconazole in children, compares them with the survey results, and outlines potential future perspectives for this compound. AML: acute myeloid leukemia; ALL: acute lymphoblastic leukemia; HCT: hematological stem cell transplantation; GVHD: Graft versus host disease; DR: Delayed release; IV: intravenous; PK: pharmacokinetic; GI: gastrointestinal; TDM: Therapeutic drug monitoring; q2w: once every 2 weeks; DDI: drug–drug interaction.

**Figure S1.** Total number of indications for isavuconazole prescriptions by center. For this item, the center could select one or more options.

**Figure S2.** Total number of centers prescribing isavuconazole for prophylaxis. For this item, the center could select one or more options.
